# Assessing the impacts of agricultural drought (SPI/SPEI) on maize and wheat yields across Hungary

**DOI:** 10.1038/s41598-022-12799-w

**Published:** 2022-05-25

**Authors:** Safwan Mohammed, Karam Alsafadi, Glory O. Enaruvbe, Bashar Bashir, Ahmed Elbeltagi, Adrienn Széles, Abdullah Alsalman, Endre Harsanyi

**Affiliations:** 1 Institute of Land Use, Engineering and Precision Farming Technology, Faculty of Agricultural and Food Sciences and Environmental Management, University of Debrecen, Böszörményi 138, 4032 Debrecen, Hungary; 2 Institutes for Agricultural Research and Educational Farm, University of Debrecen, Böszörményi 138, 4032 Debrecen, Hungary; 3 School of Geographical Sciences, Nanjing University of Information Science and Technology, Nanjing, 210044 China; 4 African Regional Institute for Geospatial Information Science and Technology, Obafemi Awolowo University, Ile-Ife, 220282 Nigeria; 5 Department of Civil Engineering, College of Engineering, King Saud University, P.O. Box 800, Riyadh, 11421 Saudi Arabia; 6 Agricultural Engineering Dept., Faculty of Agriculture, Mansoura University, Mansoura, 35516 Egypt

**Keywords:** Climate sciences, Environmental sciences, Hydrology, Natural hazards

## Abstract

This study examined the physical properties of agricultural drought (i.e., intensity, duration, and severity) in Hungary from 1961 to 2010 based on the Standardized Precipitation Index (SPI) and the Standardized Precipitation Evapotranspiration Index (SPEI). The study analyzed the interaction between drought and crop yield for maize and wheat using standardized yield residual series (SYRS), and the crop-drought resilient factor (CDRF). The results of both SPI and SPEI (-3, -6) showed that the western part of Hungary has significantly more prone to agricultural drought than the eastern part of the country. Drought frequency analysis reveals that the eastern, northern, and central parts of Hungary were the most affected regions. Drought analysis also showed that drought was particularly severe in Hungary during 1970–1973, 1990–1995, 2000–2003, and 2007. The yield of maize was more adversely affected than wheat especially in the western and southern regions of Hungary (1961–2010). In general, maize and wheat yields were severely non-resilient (CDRF < 0.8) in the central and western part of the country. The results suggest that drought events are a threat to the attainment of the second Sustainable Development Goals (SDG-2). Therefore, to ensure food security in Hungary and in other parts of the world, drought resistant crop varieties need to be developed to mitigate the adverse effects of climate change on agricultural production.

## Introduction

Rapid population increase and the growing demand for natural capital to satisfy human needs, coupled with the adverse effects of climate change is exerting tremendous burden on environmental resources around the world and it is also limiting human capacity for food production^1–3^. In spite of this, the second goal of the Sustainable Development Goals (SDGs) of the United Nations seeks to eliminate hunger and all forms of malnutrition, double agricultural productivity and generally make food sustainable and available to majority of vulnerable population by the year 2030^4–6^. The impacts of climate change however poses a major challenge to the achievement of this goal because it hampers water supply and food production in many regions across the world^7–10^ and resulting in death of nearly 25,000 people from poverty and hunger every day^11,12^.

Drought is one of the major consequences of climate change that have attracted the interest of scientists for decades. This is because drought affects millions of people around the world year after year leading to major development problems because of its dramatic effects on various aspects of human endeavors including agriculture practices and socioeconomic development^13–21^.

Europe is among the continents most affected by drought, especially in the years 1989 and 1991 when most of Europe was affected, and in 1976 when the Northern and Western parts of the continent was affected^22–24^. Drought outbreaks have been increasingly evident in many European countries leading to rising temperature and declining rainfall^25–31^, which have also resulted in economic loss to many farmers. For instance, many studies have estimated that if no climate adaptation strategies are developed and adopted in the near future, drought disruption may result in economic loss of more than €100 billion^13–15,17–19,21^. Many indices, such as the Standardized Precipitation Index (SPI)^32^; Standardized Precipitation Evapotranspiration Index (SPEI)^33^; Palmer Drought Severity Index (PDSI)^34^ and the Soil moisture deficit index (SMDI)^35^, have been developed to capture drought episodes and their characteristics. Each of these indices deals with one or more variables that influence environmental characteristics such as rainfall, temperature, river discharge, and soil moisture among others. However, SPEI and SPI are two commonly used drought indices for monitoring meteorological, agricultural, and hydrological drought. For instance, Sabau et al.^36^ noted that SPI is one of the indices that could be used for agricultural drought monitoring and yields prediction for both maize and wheat in Oradea (Romania). While Tigkas and Tsakiris^37^ recommended the Reconnaissance Drought Index (RDI) for drought mentoring on wheat yield in Greece. Labudová et al.^38^ showed that there was a good correlation between crop yield and drought indices (SPEI, SPI-2,3) in the Danubian and the East Slovakian lowlands except for potato.

Hungary is vulnerable to climate change because of the frequently occurring drought event in the country over the last few decades which has impacted many aspects of the society^39–42^. Interestingly, Gálos et al.^40^, estimated that drought conditions will be prevalent in Hungary by the end of the twenty-first century. Spinoni et al.^43^, also reported that the southern European countries, including Hungary, that have experienced many severe drought events in the last few decades, will likely experience more frequent intense drought events in the future. Studies indicate that the impacts of these drought episodes can be reduced by taking immediate steps to alleviate it^39,44–47^.

Many studies have focused on the effects of climate change because of its threat to Hungary's terrestrial habitats. In particular, the impacts of drought have been analyzed extensively in recent decades. For example, Blanka et al.^48^, Buzási^49^ and Mezősi et al.^50^ have reported the susceptibility of the Hungarian environment to climate change induced drought events. Many studies^51,52^ have confirmed that there has been a significantly lower annual rainfall in many parts of the country in recent decades especially in the summer season^53,54^, and other studies suggest that climate induced precipitation changes across Hungary is expected to continue into the future (i.e. 2070–2100), with more dry summer seasons^22,42,55,56^.

**Figure 1:**
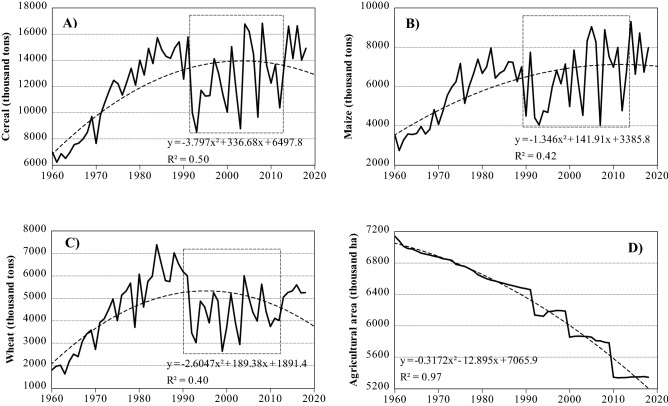
An overview of main crop production in Hungary between 1960 and 2018. (**A**) Cereal production (thousand tons), (**B**) Maize production (thousand tons), (**C**) Wheat production (thousand tons), (**D**) Agricultural lands (ha) (figure was generated using EViews (https://www.eviews.com/home.html)).

Despite the frequent incidents of drought events in Hungary, there are indications that a remarkable increase in crop yields, particularly the production of maize and wheat, have been observed in recent decades (Fig. 1). This increase in crop production has been attributed to many reasons including: (i) adopting better farming techniques (i.e., precision farming system), (ii) cultivating appropriate crop types, (iii) adequate land-use and water scheduling, and (iv) more effective pest management^57–62^. Despite the noticeable increase of crop production in Hungary, drought has a drastic impact on maize and wheat production across the country. Although some studies^63–66^ have examined the impacts of drought on agricultural production in across Hungary, there is a dearth of information on long-term regional impact of drought on crop production in Hungary. Therefore, this study seeks to: (i) evaluate the physical properties of agricultural drought (i.e., intensity, duration, and severity) in Hungary from 1961 to 2010, and (ii) analyze the interaction between drought and crop yield for maize and wheat using two indices: the standardized yield residual series (SYRS), and the crop-drought resilient factor (CDRF). This information is vital in helping scientists and decision makers gain a clear understanding of the impacts of drought events on agricultural yield. This understanding is important in providing relevant inputs for formulating strategies to mitigate the impacts of drought events on crop production.

## Materials and methods

### Study area and data collections

**Figure 2:**
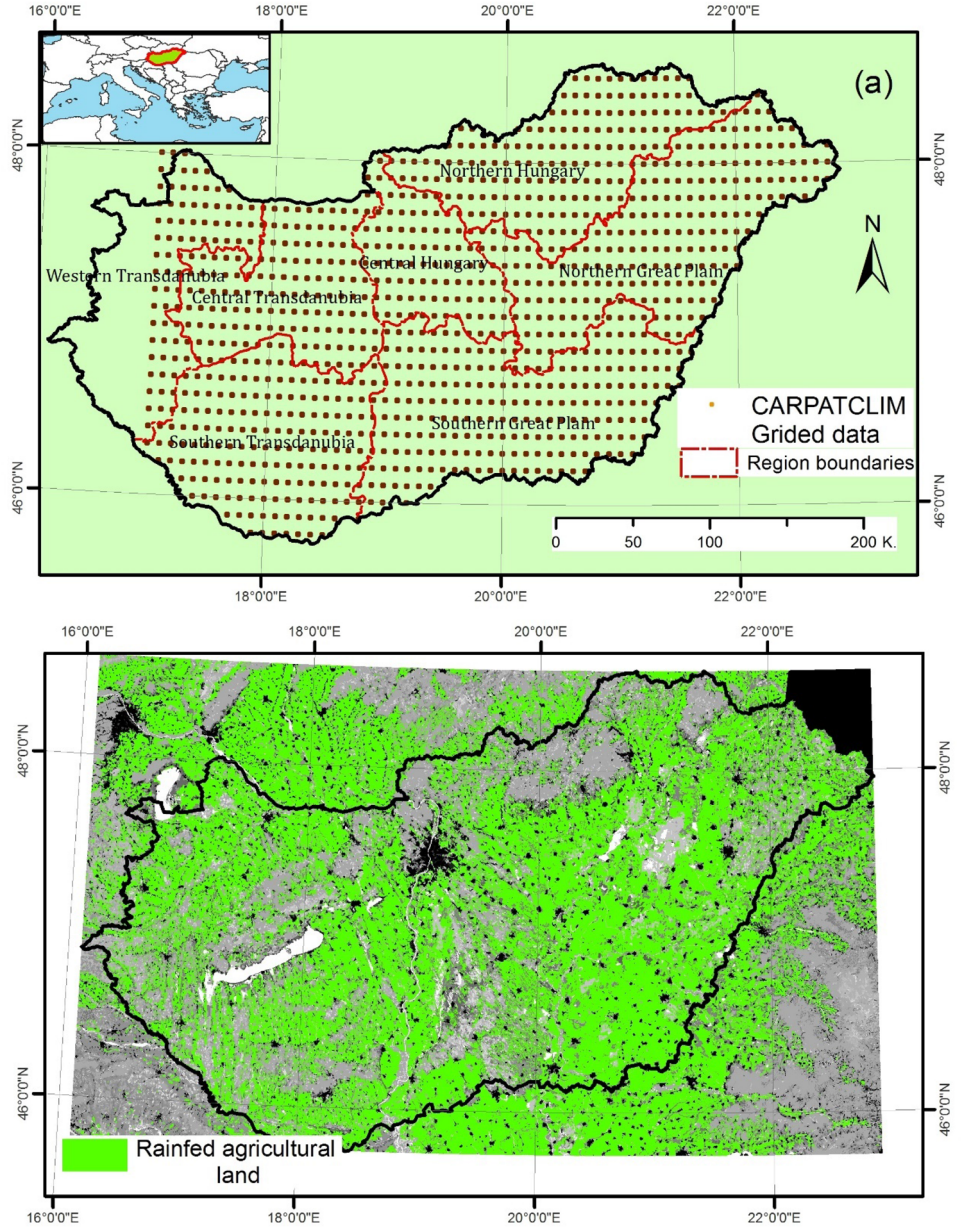
Study area (Hungary): (**a**) Distribution of gridded points across Hungary, (**b**) rainfed agricultural land (CORINE land cover data: https://land.copernicus.eu/pan-european/corine-land-cover), (maps were generated using ArcGIS 10.5 (https://www.esri.com/en-us/about/about-esri/overview)).

This research was carried out in Hungary, which is in central Europe (Latitudes 45° 55′ N–48° 60′ N and Longitudes 16° 10′ E–22° 50′ E) with a spatial extent of 93,000 km^2^ (Fig. 2). This region has a continental climate with cold and snowy winter and hot and dry summer (June, July, August). Hungary is divided into 7 regions, where each region consists of two or more counties (Fig. 2). In this context, the southern part of Hungary includes Southern Great Plain, and Southern Transdanubia, while the Central Hungary and Central Transdanubia represents the central part of Hungary. The Northern Great Plain and Northern Hungary located in northern part of Hungary, while the Western Transdanubia represents western Hungary.

Standardized precipitation index (SPI) and Standardized Precipitation Evapotranspiration Index (SPEI) recorded between 1961 and 2010 were retrieved from the database of CARPATCLIM platform (Climate of the Carpathian region project)^67,68^. The CARPATCLIM platform is an international platform which aims to improve climate data across the Carpathian Region by providing high resolution (0.1° × 0.1°) grid data of Carpathian basin. Hungary is covered by 1045 gridded points (Fig. 2). Each gridded data series provides reanalyzed data for different climate variables. In this sense, the gridded data series for SPI/SPEI on 3-, and 6-months’ time scale for the whole Hungary was downloaded. The quality and homogeneity of the CARPATCLIM data were verified and validated by the CARPATCLIM team^69^. Agricultural data for maize and wheat (e.g., cultivated area (1960–2016), total production (1960–2016), and crop yield on a regional scale (2000 to 2018) were obtained from the Hungarian Central Statistical Office (http://www.ksh.hu/).

### Standardized precipitation index (SPI) and standardized precipitation evapotranspiration index (SPEI)

Many indices were developed to capture drought events in both time and space such as SPI and SPEI. The SPI is calculated by using monthly rainfall data (20–30 years) which is subjected to a normal distribution (μ = 0, σ = 1)^70^. The structure of the SPI is based on the theory of fitting the gamma distribution to a rainfall data series. In this sense, negative SPI values indicate drought condition while a positive values refer to wet periods^71^. Notably, the SPI is recommended by the World Meteorological Organization (WMO)^82^. Similarly, the SPEI^33^ was developed somewhat from the same background as SPI, where SPEI account for climatic water balance (i.e. the standardized difference between precipitation and potential evapotranspiration). From a mathematical perspective, the SPEI integrates temperature/potential evapotranspiration into the SPI index through Thornthwaite equation^72^, which was improved by Beguería et al.^73^. More details about SPI and SPEI computation have been extensively reported in the literature^32,33^. The drought classification of both SPI and SPEI values are presented in Table 1.

**Table 1:** SPI and SPEI drought category classification^111^.

Drought group	SPI threshold	SPEI threshold
Near normal (ND)	− 1.0 ≤ SPI ≤ 1.0	− 1.0 ≤ SPEI ≤ 1.0
Moderate drought (MD)	− 1.49 < SPI < − 1.0	− 1.42 < SPEI < − 1.0
Severe drought (SD)	− 2.0 < SPI < − 1.5	− 1.82 < SPEI < − 1.43
Extreme drought (ED)	SPI ≤ –2.0	SPEI ≤ –1.83

The SPI and SPEI were obtained from CARPATCLIM platform which were derived from climate data observed in climate stations across the Carpathian Basin. The SPI and SPEI were calculated based on the shifted Gamma distribution, as its perfectly fit with the total rainfall and climatic water balance across the region^74^. While the potential evapotranspiration of SPEI structure was calculated based on the improved version of the Thornthwaite’s model^74^, which required only temperature and latitude as inputs. In this study, the SPI and SPEI at 3-months and 6-months were used to track agricultural drought across Hungary between 1961 and 2010.

### Drought trend across Hungary (1961–2010)

For detecting trend of agricultural drought across Hungary between 1961 and 2010, the Mann–Kendall (MK_t_) trend test Mann^75^ was applied. The MK_t_ is a nonparametric statistical analysis method used to assess whether the studied time series (i.e. SPI/SPEI) show an increasing or decreasing pattern^76^. One of the advantages of MK_t_ is that data do not have to align with a required distribution pattern. Therefore, extreme data values (outliers) may be included^77^. For MK_t_ the *H*_*0*_ indicates an absence of trend over time, while the *H*_*1*_ reveals a monotonic one^76^. MK_t_ and Sen’s slope were calculated for all the gridded points (1045) for the four drought indices, resulting in 8360 values (1045 × 4 × 2), which was interpolated using Geographic Information System (GIS) (ArcGIS 10.8).

### Spatio-temporal variability of drought

In order to identify agricultural drought variability pattern in space and time (i.e. spatio-temporal), across Hungary, the SPI-3, -6, and SPEI-3, -6 databases were used as input for principal component analysis (PCA)^78^. The PCA is a non-parametric method based on multivariate statistical analysis, that is widely applied in environmental science and climate research^79,80^. The PCA approach is a dimensionality reduction technique which summarizes the original data (SPI/SPEI) into a few, orthogonal, new linearly uncorrelated data that account for the majority of the variance which is referred to as Principal Components (PCs)^78,80–82^. On the basis of different combinations of variables, fixed entities, and individuals, six basic operational modes of PCA can be specified (i.e. S, Q, R, P, T, O)^78^. In this study, drought patterns (i.e., rotated loadings) were identified using the S-mode along with varimax orthogonal rotation^81^.

### Total duration of drought (*tdd*) and drought frequency in space and time

To assess the drought vulnerability, the $tdd$ (i.e., drought frequency) was computed. The $tdd$ was defined for all drought events where the SPI and SPEI are less than zero for the whole study period N_i_^83^, as denoted in Eq. (1):

1 $$tdd\left(\%\right)={n}_{i}/{N}_{i}\times 100$$

where n_i_ is the calculated drought events (less than zero), and N_i_ is the total months for time series (1961–2010). The results of $tdd$ were reanalyzed for different drought categories (i.e., to study the drought frequencies (%)) with different intensity based on Table 1. For example, a moderate $tdd$ or moderate drought frequencies (%) are computed with a calculation involving the percentage of months in which − 1 <  SPI > − 1.49 and − 1 < SPEI > − 1.42 for the whole studied period. The same process was repeated for each drought category.

The drought prone areas ($\Omega$) was identified by the percentage of the number of drought locations in the total study area (%) for each drought category, as shown in Eq. (2)^84^:

2 $$\Omega (\%)={m}_{i}/{M}_{i} \times 100$$

The $\Omega$ is the area prone to drought based on Table 1, where m_i_ is the number of gridded points when the SPI/SPEI is < 0, i is a month, and M_i_ is the whole network in the study area. The $\Omega$ method is important because it depicts the percentage of the area prone to different categories of drought.

### Impact of agricultural drought evolution on maize and wheat production

In Hungary, crop production has recently witnessed a remarkable increase (Fig. 1), because of the adaptation of modern agricultural technology. Therefore, to remove the bias attributed to non‐climatic factors, the yield data were detrended using simple linear regression model. The standardize residual from the detrended series were transformed via a z-score to capture the variability among studied crops (maize and wheat). Studies have shown that by detrending and transforming yield data using the Standardized Yield Residuals Series (SYRS) (mean = 0, standard deviation = 1)^85,86^, the effects of non-climatic factors on agricultural production can be eliminated^24,85,87,88^. The Standardized Yield Residuals Series (SYRS) was calculated using the formular in Eq. (3):

3 $${SYRS}_{cr,c,y,t}=\frac{({\xi }_{cr,c,y,t}-{\beta }_{cr,c,y,t})}{{\Omega }_{c,s,y,t}}$$

where $c:$ crop, $c:$ county, $y:$ $\mathrm{year}$, $t$ : timescale, ${SYRS}_{cr,c,y,t}$: Standardized Yield Residual Series, ${\xi }_{cr,c,y,t}:$ standardize residual from the LGM (detrended), ${\beta }_{c,s,y}^{T}$: mean of ${\xi }_{cr,c,y,t}$, ${\Omega }_{c,s,y,t}$: standard deviation of ${\xi }_{cr,c,y,t}$. The categories of the SYRS are presented in Table 2.

**Table 2:** SYRS classification^86^.

Yield	${\mathrm{SYRS}}_{c,r,y,t}$
Normal	− 0.5 < SYRS ≤ 0.5
Mild losses	− 0.5 < SYRS ≤ − 1.0
Moderate losses	− 1.0 < SYRS ≤ − 1.5
High losses	− 1.5 < SYRS < − 2.0
Extreme losses	SYRS ≤ − 2.0

To highlight the impact of agricultural drought (SPI & SPEI-3, -6) on maize and wheat production at regional scale, the crop-drought resilient factor (CDRF) was calculated for each county across Hungary. The CDRF refers to a crop's ability to withstand external stresses (such as drought) while maintaining its structure and functions^18^.

The *CDRF* was calculated following Eq. (4)^18,89,90^.

4 $$CDRF=\frac{{d}_{dr}}{{d}_{dt}}$$

Hence, ${d}_{dr}$ donates yield value in the driest year (growing cycle) during the monitoring period at regional scale, while ${d}_{dt}$ refers to detrended yield value in the same year. Table 3 shows the $\mathrm{CDRF}$ classification. To obtain the driest year, the average gridded points for SPI-3, -6 & SPEI-3, -6 values that covered each county was calculated, then the lowest value (in each county) was highlighted, then the corresponding year was chosen for CDRF calculation.

**Table 3:** Classification of the CDRF value^90^.

Crop yield resilience to drought	${CYR}_{T}$ value
Resilient	*CDRF* > 1
Slightly non-resilient	0.9 < *CDRF* < 1
Moderately non-resilient	0.8 < *CDRF* < 0.9
Severely non-resilient	*CDRF* < 0.8

### Correlation analysis between agricultural drought indices and crop yields

The relationship between drought indices and ${SYRS}_{cr,c,y,t}$ were computed to determine the impact of agricultural drought (SPI-3, SPI-6, SPEI-3, SPEI-6) on crop yields (i.e., maize, wheat). Since drought indices and ${SYRS}_{cr,c,y,t}$ (crop production) have a nonlinear relationship, the second-order polynomial regression model was used^91^. In this sense, the monthly R^2^ value between ${SYRS}_{cr,c,y,t}$ and agricultural drought (SPI-3, SPI-6, SPEI-3, SPEI-6) was calculated (2000–2010), for each region in Hungary. The maximum and minimum R^2^ values were subsequently used to produce the correlation maps using ArcGIS 10.8, to show the counties worse affected by agricultural drought.

## Results

### Agricultural drought characterization across Hungary

**Figure 3:**
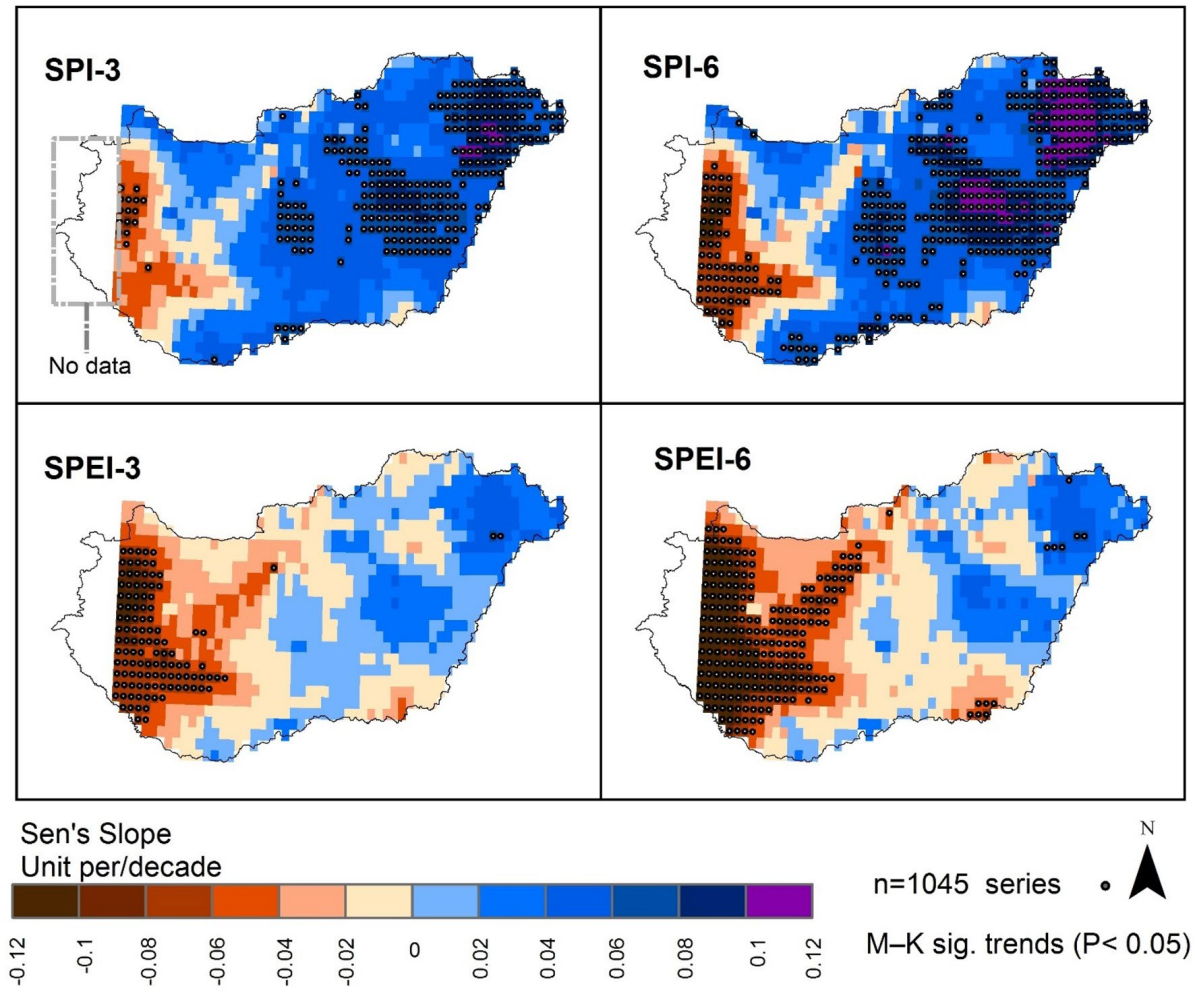
The SPI Sen's Slopes and its trends for Hungarian territory from 1961 to 2010 (maps were generated using ArcGIS 10.5 (https://www.esri.com/en-us/about/about-esri/overview)).

Similar to other extreme climate events such as flooding, drought is characterized by a temporal and spatial variation^16,44,92^. In particular, drought has been linked to poor agricultural production and water security issues especially in arid and semi-arid regions^65,93^. In this study, we examined the physical properties of agricultural drought (i.e., intensity, duration, and severity) in Hungary from 1961 to 2010 using two common drought indices at 3- and 6-months interval: SPI -3, -6 and SPEI-3, -6. Figure 3 shows that the western part of Hungary was more prone to drier conditions (i.e., drought) (brown color) than the eastern part. It also reveals that out of the 1045 gridded points for SPI-3- and -6-time scale, 16 and 72 gridded points respectively, witnessed a significant (*p* < 0.05) drought trend (MK_t_). While in the MK_t_ for SPEI-3-, and -6-time scale, 109 and 105 gridded points respectively, showed significant (*p* < 0.05) drought trend.

**Figure 4:**
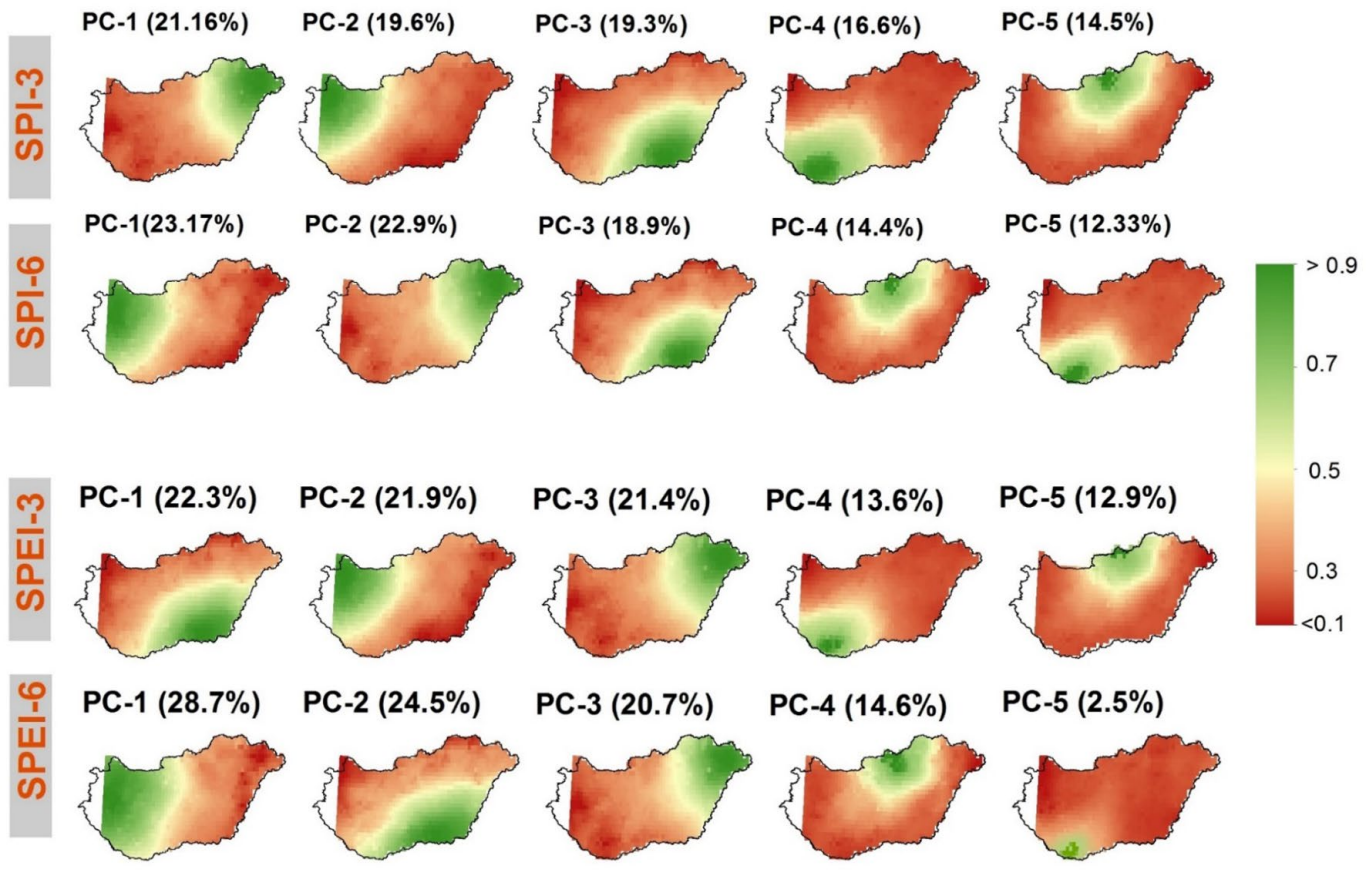
SPI-3, -6 and the SPEI-3, -6 relative to Varimax rotated loadings for the studied points (maps were generated using ArcGIS 10.8 (https://www.esri.com/en-us/about/about-esri/overview)).

Figure 4 shows the rotated loadings of the SPI-3, -6 and the SPEI-3, -6 in the study area. The figure indicates that for each drought index (i.e., SPI-3, SPI-6, SPEI-3, SPEI-6), the first five principal components (PCs) explained more than 90% of the total variance in the data. Remarkably, the first (PC1), second (PC2), and third (PC3) components explained more than 60% of the total variance of each index. The intensity and severity of drought incidences in Hungary varies over space and time as each PC is dominant in a different part of the country (Fig. 4). Although SPI-3 accounts for a minimum variance of 14% (PC5), SPI-6 accounts for 12% (PC5). In contrast, SPEI-3 accounts for maximum variance of 22.3% (Southern Great Plain) and minimum of 12.9% variance (northern Hungary). SPEI-6 accounts for a maximum of 28.7% variance (PC1, Central Transdanubia) and a minimum of 2.5% (southern Hungary).

**Figure 5:**
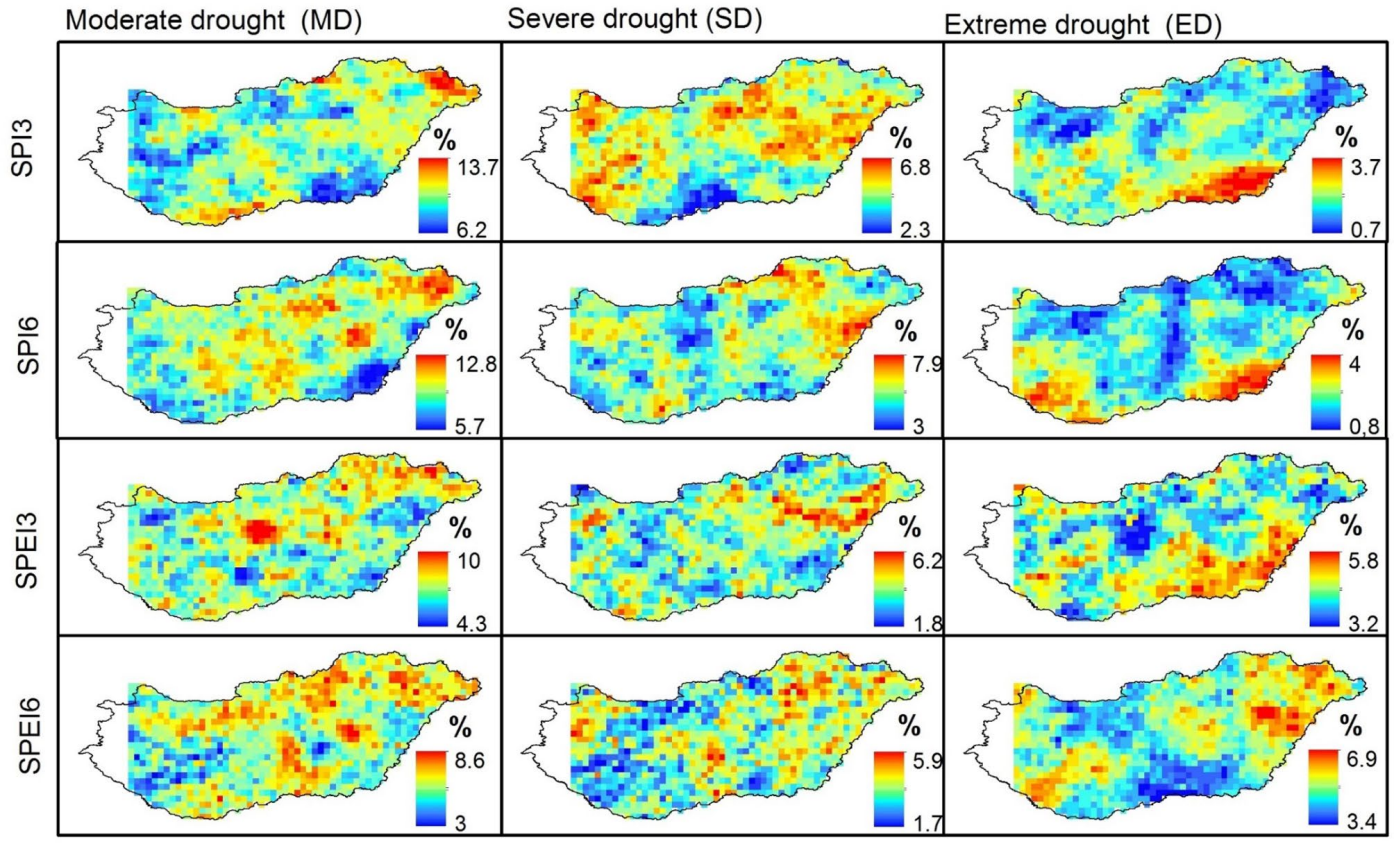
Spatial distribution of *tdd* of SPI/SPEI-3-6 between 1961 and 2010 over Hungary (maps were generated using ArcGIS 10.5 (https://www.esri.com/en-us/about/about-esri/overview)).

**Figure 6:**
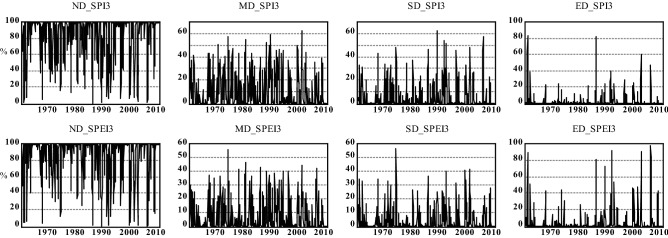
Ω(%) of different agricultural drought (SPI-3 and SPEI-3) categories between 1961 and 2010 across Hungary (figure was generated using EViews (https://www.eviews.com/home.html)).

**Figure 7:**
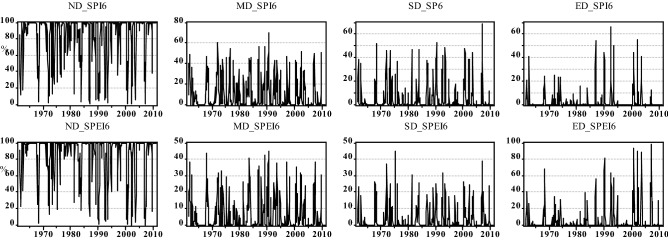
Ω(%) of different agricultural drought (SPI-6 and SPEI-6) categories between 1961 and 2010 across Hungary (figure was generated using EViews (https://www.eviews.com/home.html)).

**Figure 8:**
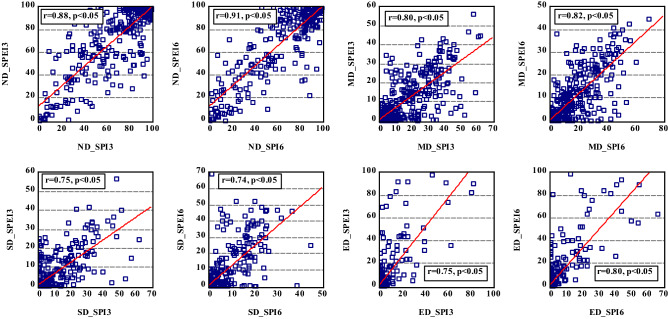
Spatial extant (%) correlation between same agricultural drought classes for SPI and SPEI between 1961 and 2010 (figure was generated using EViews (https://www.eviews.com/home.html)).

Figure 5 shows the spatial distribution of the drought frequency over Hungary based on SPI/SPEI for two different time scales (i.e., 3 months and 6 months) between 1961 and 2010. Although SPEI appears to show a more extensive agricultural drought in Hungary, agricultural drought is generally widespread in the country (Fig. 5). Figures 6 and 7 show that the common agricultural drought groups over Hungary were moderate (MD) and severe (SD) drought; and agricultural drought was severe in Hungary during 1970–1973, 1990–1995, 2000–2003, and in 2007. The MK_t_ test, however, indicates that there was no significant trend in the areas affected by different drought types except for a slight increase in areas that were not affected by drought (SPI-6) (Table 4). Regardless of the MK_t_ results, the correlation between Ω_SPI_ and Ω_SPEI_ in each category (Fig. 8) for each time scale (3-, 6-months) showed that the highest significant (*p* < *0.05*) correlation was obtained in “no drought” class (r_3-months_ = 0.88, r_6-months_ = 0.91). The second and third highest correlations were recorded in MD and ED (Fig. 8). Notably, for all drought categories, the correlation between Ω _SPI_ and Ω _SPEI_ remain significant (*p* < 0.05) and high (0.7). This indicates that the area is susceptible to drought.

**Table 4:** Trends and significance of drought proportion over Hungary between 1961 and 2010.

Drought group	SPI 3		SPEI3		SPI 6		SPEI6	
	Trend	*p*	Trend	*p*	Trend	*p*	Trend	*p*
ND	+ 1.5	0.10	+ 0.02	0.9	** + 2.12**	0.03*	+ 0.3	0.7
MD	+ 1.5	0.12	+ 0.4	0.6	+ 1.4	0.15	+ 0.6	0.5
SD	+ 1.3	0.16	+ 0.03	0.9	+ 1.2	0.2	+ 1.3	0.1
ED	+ 0.14	0.88	+ 0.5	0.5	+ 0.6	0.5	+ 1.7	0.08

*Significant p < 0.05.

Significant values are given in bold.

### Impact of agricultural drought on the yield of maize and wheat

**Figure 9:**
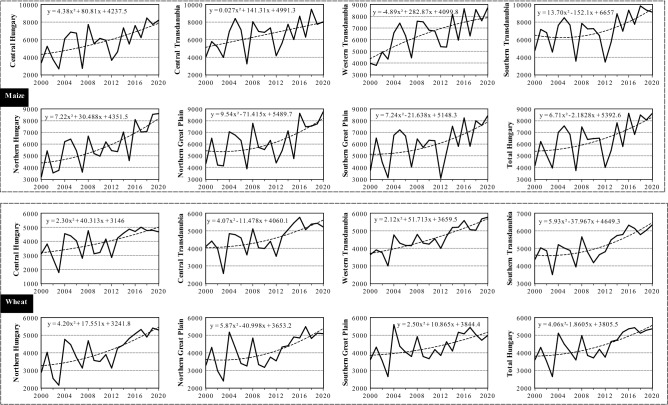
Maize and wheat yields (kg/ha) across Hungarian regions (2000–2020) (figure was generated using EViews (https://www.eviews.com/home.html)).

**Figure 10:**
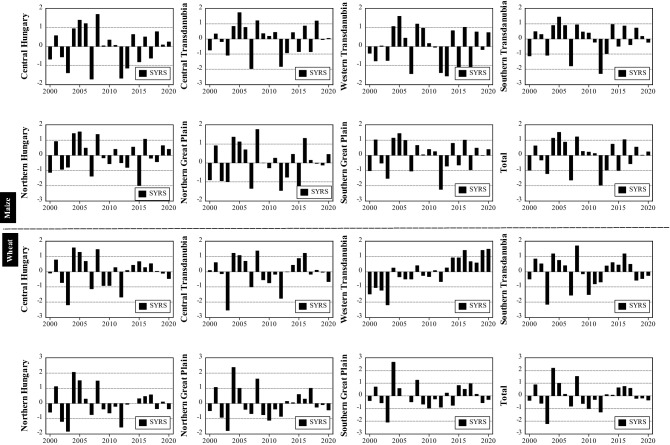
Temporal change in SYRS for both maize (up) and wheat (down) across Hungarian regions (2000–2020) (figure was generated using EViews (https://www.eviews.com/home.html)).

In this study, we analysed the interaction between drought and crop yield for maize and wheat using two indices: the standardized yield residual series (SYRS), and the crop-drought resilient factor (CDRF). Figure 9 and Table 5 indicate that in general, there was a significant (*p* < 0.05) increase in the yield of maize and wheat between 2000 and 2020. The order of yield increment for maize yield was Northern Great Plain region (+ 183.33 kg/ha, *p* < 0.05), the Central Hungary region (+ 182 kg/ha) and Western Transdanubia (+ 159.28 kg/ha, *p* < 0.05), and the order for wheat was (+ 128.56 kg/ha, *p* < 0.05) in the Northern Great Plain region, (+ 105 kg/ha, *p* < 0.05) in the Northern Great Plain and (+ 97.5 kg/ha, *p* < 0.05) in the Western Transdanubia region. However, to avoid the positive impact of new technology, fertilization and enhanced crop varieties on crop yield, the maize and wheat yield series were detrended before the SYRS was computed. Figure 10 shows the detrended value of SYRS which indicates the changes in the yield of maize and wheat across Hungarian regions, because of climate variability between 2000 and 2020.

**Table 5:** Trend (MK_t)_ and Sen's slope of maize and wheat yields across Hungarian regions between 2000 and 2020.

Region	Counties	Maize (kg/ha)	Significant	Wheat (kg/ha)	Significant
Central Hungary	Budapest + Pest	+ 182.00	0.003*	+ 76.60	0.001*
Central Transdanubia	Fejér + Komárom-Esztergom + Veszprém	+ 144.58	0.029*	+ 72.33	0.005*
Western Transdanubia	Győr-Moson + Sopron + Vas + Zala	+ 159.28	0.001*	+ 97.50	< 0.0001*
Southern Transdanubia	Baranya + Somogy + Tolna	+ 141.17	0.019*	+ 88.23	0.000*
Northern Hungary	Borsod-Abaúj-Zemplén + Heves + Nógrád	+ 183.33	0.000*	+ 128.56	0.000*
Northern Great Plain	Hajdú-Bihar + Jász-Nagykun-Szolnok + Szabolcs-Szatmár-Bereg	+ 130.33	0.013*	+ 105.00	0.002*
Southern Great Plain	Bács-Kiskun + Békés + Csongrád-Csanád	+ 125.41	0.042*	+ 68.66	0.013*
Total Hungary	–	134.68	0.0112*	93.3516	0.0006*

*Significant p < 0.05.

Between 2000 and 2020, the SYRS analysis for crop yield depicted a notable decrease (i.e., SYRS < − 1.5), namely in 2000, 2003, 2007, 2012–2013 (Fig. 10). For maize yield, the lowest SYRS value was recorded in Southern Transdanubia (− 2.29, 2012), followed by Southern Great Plain region (− 2.25, 2012), then Northern Hungary (− 2, 2015) and Central Hungary (− 1.96, 2007) (Fig. 10). On the other hand, the lowest the lowest SYRS value for wheat crop was recorded in Central Transdanubia region (− 2.54, 2003), then in Western Transdanubia region (− 2.19, 2003) (Fig. 10). Notably, the following years 2003, 2007 and 2012 witnessed a direct impact of climate on maize yield losses, while 2003 was a drastic year for wheat production in Hungary. It is good to mention here, according to SYRS analysis the wheat yield was less exposure and affected by drought episodes in comparison with maize. As such, the wheat crop appears to be more drought resistant than maize across Hungary.

#### Drought impact on maize and wheat yield

**Figure 11:**
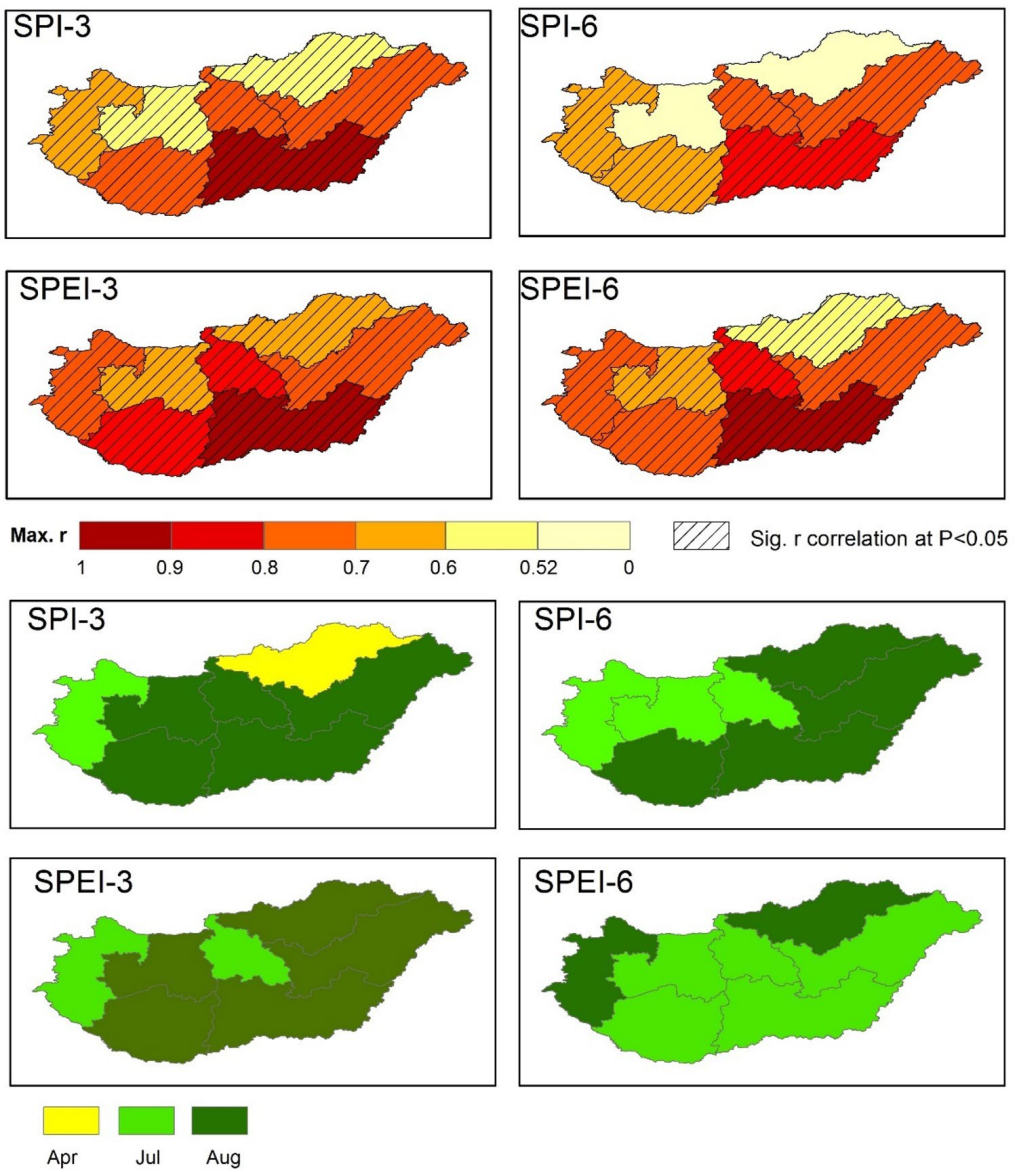
Spatial distribution of maximum correlation between drought indices (SPI-3, SPEI-3, SPI-6, SPEI-6) and SYRS for maize across Hungary (2000–2010) (upper figures), the corresponding month where the maximum correlation is occurred during the growing cycle (lower figures) (maps were generated using ArcGIS 10.5 (https://www.esri.com/en-us/about/about-esri/overview)).

**Figure 12:**
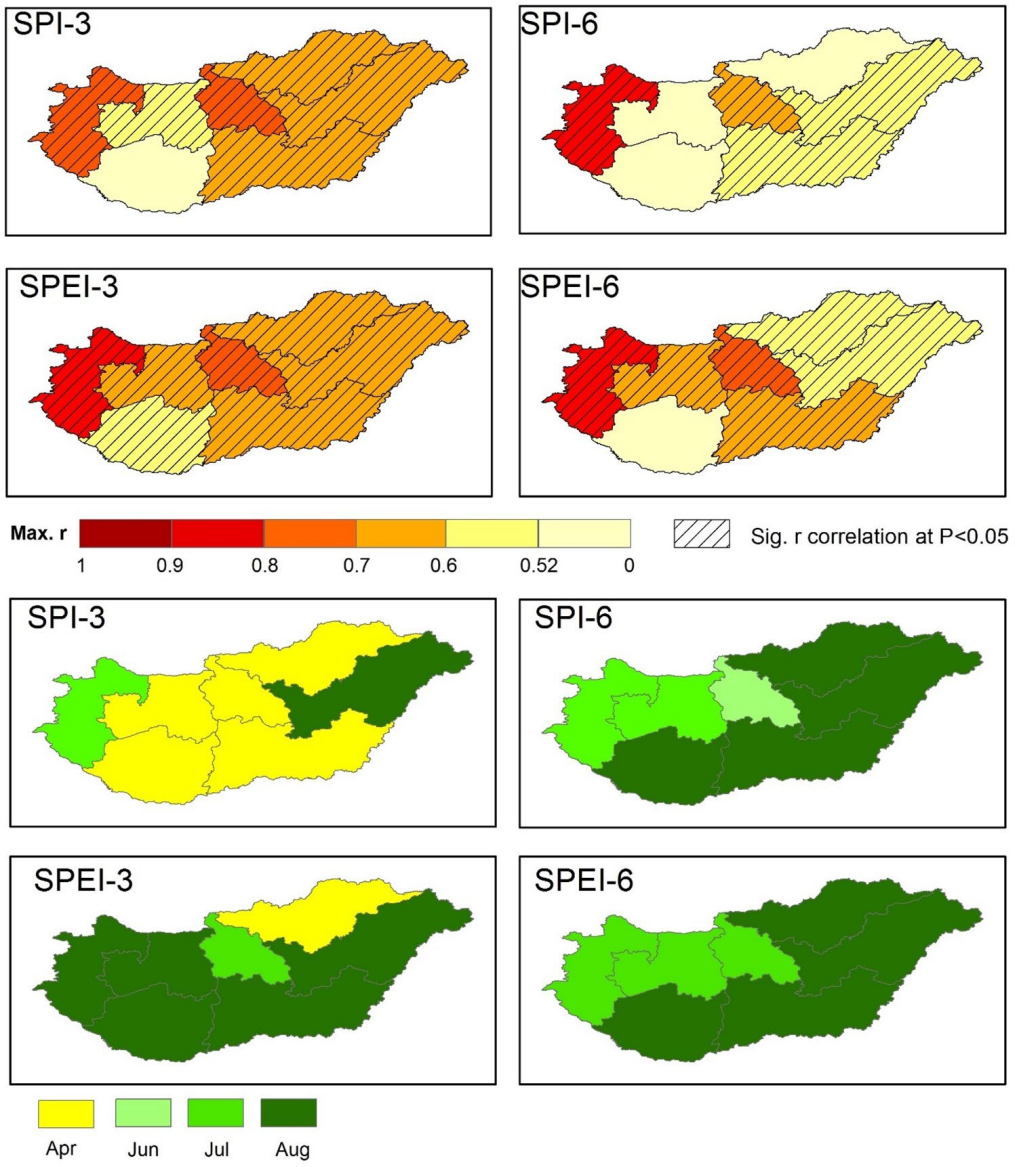
Spatial distribution of maximum correlation between drought indices (SPI-3, SPEI-3, SPI-6, SPEI-6) and SYRS for wheat across Hungary (2000–2010) (upper figures), the corresponding month where the maximum correlation is occurred during the growing cycle (lower figures) (maps were generated using ArcGIS 10.5 (https://www.esri.com/en-us/about/about-esri/overview)).

To capture the direct impact of drought spells on crop yield across different regions of Hungary during the period of this study, we determined the Pearson correlation coefficients (*r*) between drought indices (SPI-3, SPEI-3, SPI-6, SPEI-6) and the SYRS for wheat and maize on a monthly scale. However, because the available data for drought indices was available from 1961 to 2010, and the crop production data across Hungarian regions was limited to 2000–2020, we restricted our analysis to the intersecting years between them (i.e., 2000–2010). Our analysis suggests that crop yield is susceptible to drought events especially during the growing cycle, particularly in August. Where, the highest correlation during the growing cycle occurred in June and August over most Hungarian regions for maize and wheat (Figs. 11 and 12). However, an extensive analysis should be carried out as new data become available.

The highest correlation between SPI-3 and SYRS (*r*_*SPI-3vs.SYRS*_) for maize (0.92 (*p* < 0.05)) was observed in the Southern Great Plain region, followed by Northern Great Plain and Southern Transdanubia (*r*_*SPI-3vs.SYRS*_ = 0.75, *p* < 0.05) (Fig. 11). Similarly, for SPEI-3 the highest *r*_*SPEI-3vs.SYRS*_ was observed in the Southern Great Plain region (0.92, *p* < 0.05), and in Central Hungary (0.8, *p* < 0.05). In terms of 6-month, the *r*_*SPI-6vs.SYRS*_ in Southern Great Plain (*r*_*SPI-6vs.SYRS*_ = 0.90, *p* < 0.05), and Central Hungary regions (*r*_*SPI-6vs.SYRS*_ = 0.74, *p* < 0.05) was the highest one among other regions (Fig. 11). Like the SPI-6, the highest correlation between SPEI-6 and the SYRS (*r*_*SPEI-6vs.SYRS*_) was in Southern Great Plain (*r*_*SPI-6vs.SYRS*_ = 0.92, *p* < 0.05), and Central Hungary regions (*r*_*SPI-6vs.SYRS*_ = 0.85, *p* < 0.05).

The highest correlation between SPI-3 and SYRS (*r*_*SPI-3vs.SYRS*_) for wheat was recorded in Western Transdanubia and Central Hungary regions (*r*_*SPI-3vs.SYRS*_ = 0.74, *p* < 0.05) (Fig. 12). Like SPI-3, the highest *r*_*SPEI-3vs.SYRS*_ was also recorded in Western Transdanubia and Central Hungary regions (*r*_*SPEI-3vs.SYRS*_ = 0.87, *p* < 0.05). We observed consistently high correlation between drought indicators (SPI-6 and SPEI-6) and crop yield in Western Transdanubia and Central Hungary regions (Fig. 12). These results emphasis the direct impact of drought episodes on crop production across Hungary.

**Figure 13:**
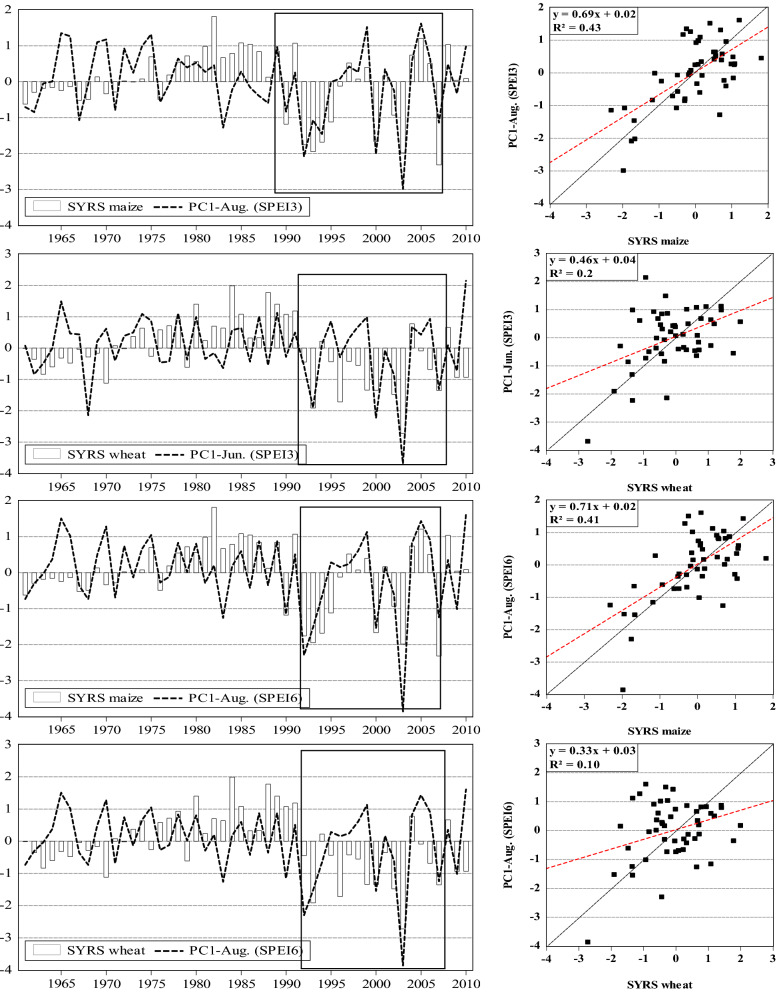
Drought loading (PC1) vs. SYRS for maize and wheat across Hungary (1961–2010) (figure was generated using EViews (https://www.eviews.com/home.html)).

As the above analysis were limited to 11 years due to data scarcity, we extended our analysis to a national scale by linking drought loading (i.e., PC1, PC2, ….) (Fig. 4) with detrended national yield (SYRS) of maize and wheat yield between 1961 and 2010. The results at the National scale, show that maize yield was more adversely affected by drought (${r}_{\mathrm{PC}1,\mathrm{ SYRS}}^{2}$=0.43) than the yield of wheat (${r}_{\mathrm{PC}1,\mathrm{ SYRS}}^{2}$=0.21) (Fig. 13). Figure 13 shows that the impact of agricultural drought is widespread in Hungary. This was particularly severe across the country between 1990 and 2007 (Fig. 13). The other PCs (i.e., PC2, PC3, PC4, PC5) were however neglected as the ${r}_{\mathrm{PC}1,\mathrm{ SYRS}}^{2}$ did not exceeded 0.09.

#### Crop resilience to drought (*CDRF*) on a regional scale of Hungary

**Figure 14:**
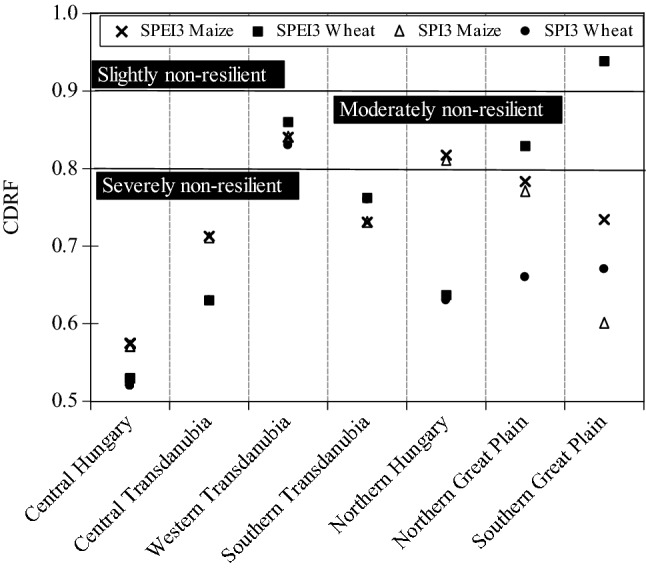
Crop drought resilience factor ($CDRF$) in terms of 3 months’ time scale for maize and wheat across Hungarian regions between 2000 and 2010 (figure was generated using EViews (https://www.eviews.com/home.html)).

The crop-drought resilient factor (CDRF) was calculated across Hungary for the period 2000–2010, on a regional basis. On 3 months’ timescale, only wheat yield in Southern Great Plain region was slightly affected by drought (CDRF_SPEI-3_ = 0.94) (Fig. 14). Wheat yield in Northern Great Plain region (CDRF_SPEI-3_ = 0.83), as well as Maize yield in Northern Hungary region (CDRF_SPEI-3_ = 0.81, CDRF_SPI-3_ = 0.81) were depicted a moderate resistance to drought. In Western Transdanubia region maize and wheat yield was moderately affected by drought (Fig. 14). However, the CDRF analysis in the rest regions indicate that crop yield was severely non-resilient, whereas the lower CDRF value was recorded in Central Hungary region (CDRF_SPEI-3_ = 0.52).

**Figure 15:**
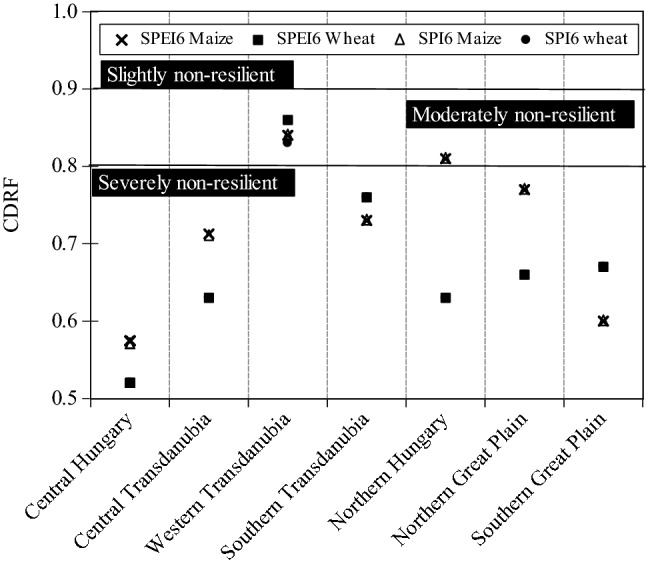
Crop drought resilience factor ($CDRF$) in terms of 6 months’ time scale for maize and wheat across Hungarian regions between 2000 and 2010 (figure was generated using EViews (https://www.eviews.com/home.html)).

Figure 15 depicted the resistance of maize and wheat to drought episodes in 6-month interval. Majority of the regions exhibited that crop yield was severely non-resilient to drought (CDRF < 0.8), except for Northern Hungary, and Western Transdanubia. Noticeably, the lowest value was captured in Central Hungary region (CDRF_SPEI-6_ = 0.52).

All in all, the CDRF analysis for each drought index (i.e., SPI, SPEI) indicate that crop yield was badly affected by drought in most of Hungarian regions which drew the attention to the importance of regional climate adaptation measures to minimize the drought impact on crop production.

## Discussion

Agricultural drought remains a major threat to food security in many parts of the world. This is particularly severe in arid and semi-arid regions of the world^93^. Recently, drought is becoming more severe in many parts of central Europe, causing concerns about its impacts on agricultural productivity generally and especially on crop yield^94^. The results of this study show that although agricultural drought is widespread in Hungary, it ranges from moderate to severe and may depend on the interval and methods used in determining drought events. Drought was particularly severe during 1970–1973, 1990–1995, 2000–2003 and in 2007 (Figs. 6 and 7). In addition, the western part of Hungary was more prone to dry conditions than other parts of the country (Fig. 3). The difference values observed between SPEI and SPI may be because of the differences of input and method of calculations^38,86,95^. It is good to mention here that both indices have positive and negative points. The weak points of the SPI are that it depends only on the precipitation data with the total neglect of precipitation distribution over time. On the other hand, using only temperature for calculating PET through Thornthwaite equation considered to be one of the main disadvantages of the SPEI. In other words, calculating PET through different method could lead to a different result Alsafadi et al.^22^.

We observed that drought events were frequent in the central part of Hungary (i.e., Great Hungarian Plain) (Fig. 5). Similar finding was reported by Alsafadi et al.^22^ and Farkas and Török^96^. The low rainfall in the central part of the country may be related to the warmer and drier climate conditions over Hungary since the beginning of the present century^97^. For instance, while SPI-3 indicates dominate drought pattern in the western part of the country, also, SPI-6 shows dominant drought variance in the western part (Figs. 3 and 4). The spatial variation in the occurrence of drought in Hungary may be related to the pattern of rainfall in the country which may be explained by several factors including: (i) increase of solar/geomagnetic forces which amplify the drought impact especially in summer. For instance, Mares et al.^98^ noted a significant correlation between drought index and solar/geomagnetic in central Europe, and (ii) changes in the continental factors in the Great Hungarian Plain which affected rainfall patterns and altered the ecosystem. For instance, Mohammed et al.^99^, asserted that rainfall pattern in Hungary led to wide variability of drought events in Eastern Hungary. However, according to the SPEI westeren part of Hungary appears to be susiptable to drought compering with the output of the SPI. This could be because of the computation of SPEI accounts for temprature and evapotransperation along with rainfall but SPI accounts for only rainfall.

Maize is one of the staple foods crops and it occupies 8.9–51.1% of cultivated area in Hungary^100^, which is the fourth largest producer in the world^101^. Although, water requirements for crop such as maize, can be provided by rainfall during growing cycle, the impact of agricultural drought across Hungary have been reported to adversely affected crop yield^65,102^. The results of this study indicate that crop yield was severely non-resilient to drought (i.e., CDRF < 0.8) (Figs. 14 and 15). In addition, we observed that crop yield is susceptible to drought events especially during the growing cycle. This finding confirms the assertion by Fiala et al.^65^ who reported that maize losses are highly correlated with drought severity. Interestingly, Adrienn and Janos^102^ reported that water stress is one of the main factors influencing the decline in crop production in Hungary. Juhász et al.^103^ has shown that the changing pattern of rainfall could have resulted in more than 25% losses in crop yield across Hungary between 2003 and 2013. The Hungarian agricultural system mostly rainfed and therefore climate vagaries such as rainfall deficit could lead to severe crop failure and promote food security challenges^104^. The western part of Hungary has experienced substantial reduction in rainfall trends^105^, along with increasing drought frequency during summer seasons which may have impaired crop production in recent decades.

Many studies such as Lobell et al.^106^ and Webber et al.^107^, have shown the sensitivity of maize to drought. However, the crop-drought resilient factor (CDRF) shows that maize may be less resilience to drought than wheat (Figs. 14 and 15). We observed a positive correlation between drought indices and SYRS during the growing cycle for maize and wheat suggesting that these crops are susceptible to drought events, especially in the tasseling stage (TS) of maize. Adrienn and Janos^102^ has reported that drought stress, especially during the tasseling stage, reduced the yield of maize by as much as 50%. Fiala et al.^65^ also reported a strong link between drought severity and maize yield loses. Similar conclusions have been reach by Daryanto et al.^108^ who noted that maize is more sensitive to drought than wheat, especially in reproductive phase. In this context, the divergence in the response of wheat and maize to drought stress can be mainly attributed to their origins. Whereas, the origin of wheat is dray regions^109^, while the wet region is the origin of maize^110^. Therefore, the inherent adaptability characteristics may play a key role in crop adaptation^108^.

This paper reports that agricultural drought is more likely to occur over Hungary. The results of this study indicate that mild to moderate drought condition is a common type of agricultural drought over Hungary. As the SPI index only used rainfall data, and the SPEI index used rainfall and temperature data, our next step will be to try to track vegetation cover changes by using the Normalized Difference Vegetation Index (NDVI) to understand the interaction between drought and vegetation cover. Nonetheless, Mohammed et al.^99^ reported a significant correlation between SPI-3, -6 index and NDVI during the summer season in the eastern part of Hungary.

Although irrigation could be one of the possible solutions to minimize the direct impact of drought on crop production, especially in central and southern part of Hungary, economic considerations limits it as an option because of the more than 500 thousand hectares of the Hungarian agricultural land^101^. Therefore, a mitigation plan that minimize the potential impact of drought on agricultural production, taking into consideration a combination of three important elements: (1) soils, (2) climate, and (3) cropping patterns should be developed. On the other hand, the interaction between different agroecosystems in Hungary (i.e., maize, wheat, apple, etc.) and drought characteristics (duration, time) should be carefully evaluated and associated with crop improvement (e.g., cultivating drought-tolerant crops varieties) and proper land management.

The output of this research will help in better understanding of agricultural drought evolution across Hungary (1961–2010) in both space and time. Based on the output of this research, future development plans could be oriented toward supporting areas that have more susceptibility to agricultural drought (i.e., central, and western parts). Also, decision-makers could develop national plans for climate adaptation and mitigation with a special focus on the vulnerable areas in accordance with EU policies such as the common agricultural policy (CAP).

## Conclusion

Climate change poses a significant threat to human wellbeing and food security especially in arid and semi-arid regions of the world. In this study we examined the physical properties of agricultural drought such as intensity, duration, and severity in Hungary from 1961 to 2010, and analyzed the interaction between dryness and crop yield for maize and wheat using standardized yield residual series (SYRS), and the crop-drought resilient factor (*CDRF*). We observed that extreme climate events are becoming more frequent in Hungary over the years. Our study highlighted the exposure of the western part of Hungary to drought, where the drastic years were in 1970–1973, 1990–1995, 2000–2003, and 2007. The western and Eastern regions of Hungary are more prone to extreme climate events. We observed that the impact of drought is more severe on maize production than on wheat. However, maize and wheat were severely non-resilient to drought (${CYR}_{T}$< 0.8) in most counties across Hungary. Our results demonstrate the need to develop drought resistant crop varieties to mitigate the impacts of extreme climate events in the arid and semi-arid regions of the world. Although this study has shown the influence of drought events on agriculture, we recommend that further studies should explore the climate change impacts on agriculture using other drought indices including Palmer drought severity index (PDSI) and precipitation evapotranspiration difference condition Index (PEDCI). It is likely that a comparison of these indices will enhance our understanding of agricultural drought events.

## Data Availability

Climate data: http://www.carpatclim-eu.org/pages/home/, crop production in Hungary: http://www.ksh.hu/.
